# Temporary Fixation of Reduction with Fabric Adhesive Bandage in the Surgical Treatment of Pediatric Supracondylar Humerus Fractures

**DOI:** 10.3390/medicina55080450

**Published:** 2019-08-07

**Authors:** Ozan Turhal, Mustafa Kınaş, Zekeriya Okan Karaduman, Yalçın Turhan, Onur Kaya, Cemal Güler

**Affiliations:** 1 Department of Orthopaedics and Traumatology, Duzce State Hospital, Düzce 81000, Turkey; 2 Department of Orthopaedics and Traumatology, Bandırma Royal Hospital, Balıkesir 10000, Turkey; 3 Department of Orthopaedics and Traumatology, Duzce University, Medical Faculty, Düzce 81000, Turkey; 4 Department of Orthopaedics and Traumatology, Cizre State Hospital, Şırnak 73200, Turkey; 5 Department of Orthopaedics and Traumatology, Çorum Hitit University, Medical Faculty, Çorum 19000, Turkey

**Keywords:** supracondylar humerus fractures, fluoroscopy-guided reduction and fixation, fabric adhesive bandage

## Abstract

*Background and objectives:* Supracondylar humerus fractures are common in children and can be surgically treated. However, the general surgical procedures involving reduction and fixation might lead to reduction loss, failure to direct the Kirschner (K)-wire toward the desired position, prolonged surgery, or chondral damage. This study aimed to show that temporary fixation of closed reduction with a fabric adhesive bandage in pediatric supracondylar humerus fractures could maintain reduction so that surgical treatment can be easily performed by a single physician. *Materials and Methods:* Forty-six patients with Gartland type 3 supracondylar humerus fractures who underwent surgical treatment between May 2017 and June 2018 were retrospectively evaluated. Fluoroscopy-guided reduction and fixation were performed from the distal third of the forearm to the proximal third of the humerus using a fabric adhesive bandage. Two crossed pins were applied on the fracture line by first inserting a lateral-entry K-wire and then inserting another K-wire close to the anterior aspect of the medial epicondyle and diverging from the ulnar nerve tunnel. A tourniquet was not applied in any patient and no patients required open reduction. *Results:* The study included 32 boys (69.6%) and 14 girls (30.4%) (mean age, 7.1; range, 2–16 years). The mean hospital stay and follow-up duration were 4.3 ± 3.9 days and 48.1 ± 14.3 weeks, respectively. Heterotopic ossification was detected in one patient, and ulnar nerve neuropraxia was detected in another patient. Functional (according to Flynn criteria) and cosmetic outcomes were excellent in 95.6%, moderate in 2.2%, and poor in 2.2% of patients. The mean duration of fixation of the closed reduction with a fabric adhesive bandage was 8.1 ± 3.9 min, and the mean duration of pinning was 7.9 ± 1.4 min. *Conclusions:* Temporary preoperative fixation of supracondylar humerus fractures that require surgical treatment with a fabric adhesive bandage may be significantly convenient in practice.

## 1. Introduction

Supracondylar humerus fractures are extra-articular fractures that pass through the olecranon fossa and encompass the distal humeral condyles; they are considered distal humerus fractures and the second most common fracture type in children with distal radius fractures [1,2]. Although fractures around the elbow are the second most common traumatic fractures in children, supracondylar fractures constitute the majority (85%) of fractures that require surgical treatment [3,4]. These fractures are generally encountered in boys aged 3–10 years.

Supracondylar humerus fractures are of great significance as their conservative or surgical treatment could have an effect on the patient’s future lifestyle; these fractures also affect hand, wrist, and forearm movements. Therefore, their treatment is very important to prevent future functional and cosmetic morbidities [5,6]. The treatment of supracondylar humerus fractures, which occur in the neurovascular region and due to trauma or iatrogenic causes, requires sufficient experience and skill [5].

The mechanism of injury in supracondylar humerus fractures generally involves falls on an open hand with an extended elbow; however, falls with a flexed elbow do occur in 1–2% of patients [7]. Although surgical treatment varies depending on the experience of the surgeon, surgery is essential for Gartland type 3-4 fractures [8]. During surgery for supracondylar humerus fractures, one person performs reduction and tries to maintain the position, while another person carries out fixation using a Kirschner (K)-wire. The fact that at least one person is needed to ensure that the reduction is maintained during fixation with the K-wire without having the required angle of view while inserting the K-wire, as the pediatric supracondylar surgery site is narrow, could lead to a loss of reduction, not being able to direct the K-wire toward the desired position, iatrogenic nerve injury, prolonged surgery, and chondral damage [9]. Therefore, this study aimed to investigate supracondylar humerus fracture fixation using a fabric adhesive bandage following fluoroscopy-guided closed reduction under anesthesia to reduce the need for percutaneous pinning by a single physician and decrease the duration of surgery. 

## 2. Materials and Methods

The study design was approved by the Düzce University Clinical Research Ethics Committee (Düzce, Turkey) (No. 2019/170 from 5 August 2019), and the study was performed in accordance with the principles of the Declaration of Helsinki. Informed consent was obtained from the parents/guardians of the patients included in the study.

A total of 46 patients who underwent closed reduction followed by fixation using a fabric adhesive bandage and application of crossed K-wires between May 2017 and June 2018 due to Gartland type 3 pediatric supracondylar fractures were retrospectively evaluated from clinical, radiological, and functional aspects. This study evaluated the results of patients who underwent surgical treatment under general anesthesia provided by surgeons experienced in pediatric trauma at two different centers. Patients who had a fully developed skeletal system, who underwent open reduction, or who had preoperative neurovascular injury were excluded from the study.

Patient demographics, age, side of the affected extremity, follow-up duration, plaster fixation duration, pinning duration, and complications were evaluated. A functional assessment was performed to determine the flexion, extension, and internal and external rotation angles of both elbows. The difference between the intact elbow and operated elbow was evaluated by measuring and adding the flexion and extension values. In the cosmetic assessment, carrying angles were measured in both arms with a goniometer using the McRae method [10]. The difference between the two values was considered the carrying angle loss. Radiological evaluation was performed by measuring Baumann’s angle. According to the Flynn criteria, a value between 0° and 5° for the difference in the range of motion between the intact elbow and the operated elbow was considered functionally excellent, whereas a value between 6° and 10° was considered good, 11° and 15° was considered moderate, and >15° was considered poor (Table 1). A tourniquet was not applied to any of the patients. 

### 2.1. Surgical Technique

**Figure 1:**
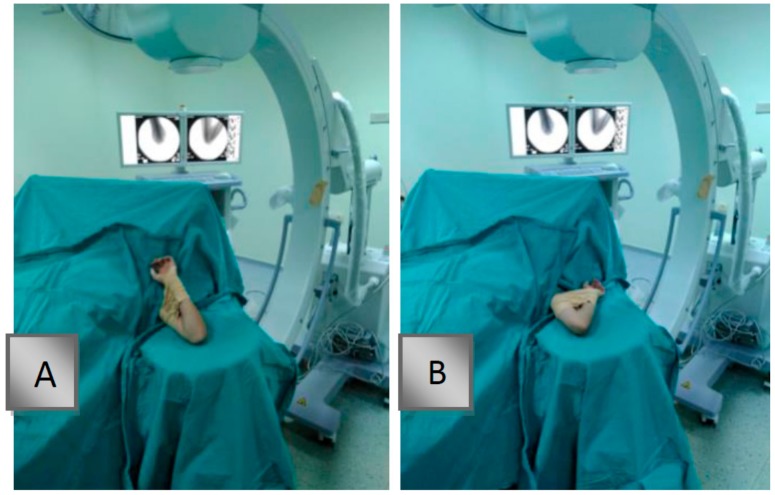
Temporary fixation of reduction with a fabric adhesive bandage. (**A**) An anteroposterior position image on fluoroscopy. (**B**) Lateral position image on fluoroscopy.

The patients were under general anesthesia and placed in a supine position on the operating table. The elbows were then placed on the fluoroscope in a manner that allowed fluoroscopic imaging. The fluoroscopy screen was positioned in front of the surgeon performing the reduction so that the surgeon could check the stages of reduction. Longitudinal traction was applied in extension, and care was taken not to stay in the varus and valgus positions with the assistance of fluoroscopy. The elbow was placed in flexion with the forearm in pronation, and opposite forces were then applied on the fracture line with the thumb on the olecranon and other fingers on the proximal aspect. The posterior and anterior views of reduction were evaluated in the Jones position, and the lateral view was evaluated with the shoulder in external rotation. Fixation was performed from the distal third of the forearm to the proximal third of the humerus using a fabric adhesive bandage (Figure 1). The fabric adhesive bandage was removed after being soaked with alcohol following the operation. None of the patients had skin irritation.

**Figure 2:**
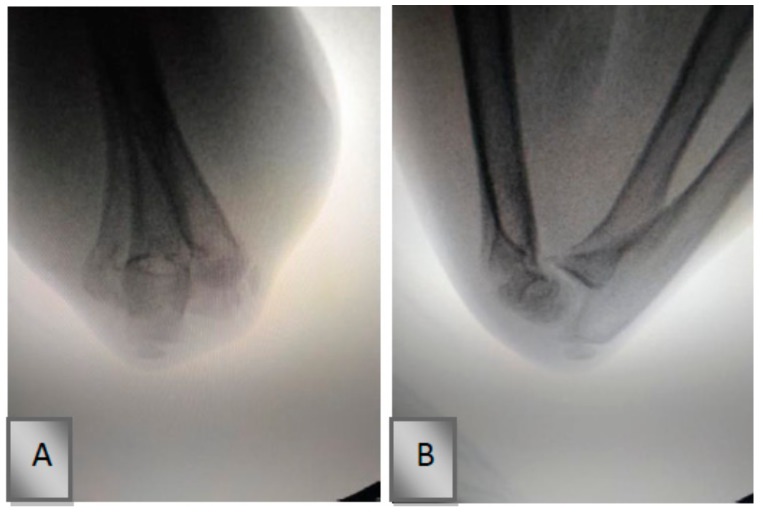
Fluoroscopic image of the supracondylar fracture after fixation with a fabric adhesive bandage. (**A**) Anteroposterior image and (**B**) lateral X-ray image of the left elbow.

The final state of reduction was re-evaluated, and anterior-posterior and lateral radiographs were obtained (Figure 2). The area distal to the fabric adhesive bandage fixation site was disinfected using Batticon, and the sterile surgical site was then prepared. The surgical site was again positioned on the fluoroscope. A 1.8- or 2.0-mm K-wire was inserted in an oblique configuration to reach the proximal medial cortex of the fracture line through the lateral condyle. Thereafter, the skin on the medial epicondyle was moved posteriorly as much as possible by moving away from the ulnar nerve tunnel. The initial insertion point of the K-wire was determined using fluoroscopy in the anterior-posterior and lateral positions. Fluoroscopy was used to ensure that the K-wire did not go into the ulnar nerve tunnel until passing the first cortex. Then, the K-wire was moved past the lateral cortex from the proximal level of the fracture line. K-wires were stopped right after passing the cortex level to prevent radial nerve and soft tissue irritation.

**Figure 3:**
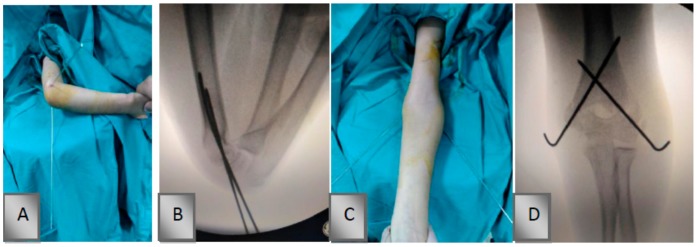
Crossed Kirschner (K)-wire configuration and its fluoroscopic image. (**A**,**C**) Anteroposterior and lateral positions after K-wire configuration and (**B**,**D**) intraoperative radiograph with crossed K-wire configuration.

After the crossed pin configuration, sterile drapes and the fabric adhesive bandage used in the fixation method were removed. Lateral radiographs of the elbow were obtained with the shoulder in external rotation, whereas anterior-posterior images were obtained in extension (Figure 3).

Joint range of motion was evaluated after removing the fabric adhesive bandage. The K-wires were confirmed to have been placed close to the anterior aspect so that they would not prevent elbow extension. Then, the surgery was terminated after placing a long arm splint at 70–90° flexion and pronation. The surgical site was elevated, and ice packs were applied during patient follow-ups. Edema and blood circulation were routinely evaluated.

**Figure 4:**
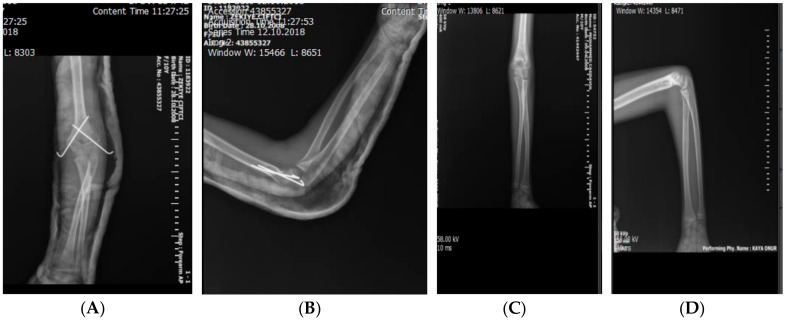
Direct radiographs obtained in the preoperative period and on day 90. (**A**,**B**) Postoperative X-ray image showing that the fracture was fixed by two K-wires and (**C**,**D**) anteroposterior and lateral views after removal of K-wires three months postoperatively.

Direct radiographs were obtained on postoperative days 1, 15, and 30 and again on day 45 after removing the K-wires (Figure 4). The long arm splint was removed on day 20, and passive and active exercises were started. K-wires were removed between days 35 and 45 depending on the patient’s age and union status. None of the patients needed rehabilitation. The final follow-up was performed at six months to obtain bilateral elbow radiographs and perform functional and cosmetic assessments.

### 2.2. Statistical Analysis

The SPSS 23.0 software package (SPSS Inc., Chicago, IL, USA) was used for statistical analysis. Categorical measurements were summarized with numbers and percentages and continuous measurements with mean and standard deviation values (median and minimum-maximum where necessary). The dependent group *t*-test was used for the comparison of the intact side with the fractured side. The statistical significance level was *p* < 0.005 for all tests.

## 3. Results

Overall, 46 patients were included in the study. The mean age was 7.1 ± 3.4 (2–16) years; 69.6% of patients were boys and 30.4% were girls, and 56.5% of patients had a fracture on their right side. For all surgeries, 1.8- or 2.0-mm K-wires were used. Thicker wires were used in parallel with increased patient age and bone diameter to enhance stability. The patients were followed for a mean period of 46 (20–82) weeks. The mean duration of reduction and plaster fixation was 6.5 min, and the mean duration of pinning was 8 min. At the six-month follow-up, the mean range of motion (ROM) difference between the intact elbow and operated elbow was 0.59 ± 2.8°, which was not statistically significant (Table 2).

In this study, nine children developed fractures due to a simple fall in the house, 35 children had fractures due to a fall while playing in the park, and one developed fracture due to a traffic accident. According to the Flynn criteria, functional outcomes were excellent in 95.6% of the patients, moderate in 2.2%, and poor in 2.2%, whereas cosmetic outcomes were excellent in 95.6%, moderate in 2.2%, and poor in 2.2% of the patients (Table 3). The patient with a supracondylar fracture due to a traffic accident also had a non-displaced distal radius and ulnar fractures and he did not need any surgical treatment for these additional fractures. 

According to the direct radiography images at six months, there was no statistically significant difference between the intact side and the side with the fracture in terms of Baumann’s angle (*p* = 0.069). There was no statistically significant difference between the carrying angles of the intact side and the side with the fracture based on the follow-up radiographs at six months (*p* = 0.303) (Table 4).

## 4. Discussion

We found that supracondylar humerus fractures were more prevalent in boys. In addition, the fabric adhesive bandage fixation method following fluoroscopy-guided closed reduction under anesthesia was considered safe for the ulnar nerve, and it provided sufficient biomechanical stability. 

Pediatric supracondylar fractures are generally encountered in children aged 3–10 years. In the studies of Gosens et al. and Erdil et al., the mean patient age was 7.7 years and 6.2 years, respectively [11,12]. In the present study, the mean patient age was six (2–16) years, which is consistent with that in the literature. Furthermore, evaluation of patients according to sex showed that supracondylar humerus fractures were more prevalent in boys [11,12,13,14]. Our study also showed a higher prevalence in boys as the distribution of the patients by sex was as follows: 69.6% boys and 30.4% girls. The length of hospital stay in our study was 4.3 ± 3.9 days, which is consistent with that in the literature [15,16].

The current treatment recommendations for supracondylar humerus fractures are based on the Gartland classification of the American Academy of Orthopedic Surgeons [17,18]; surgery is essential for Gartland type 3–4 injuries. During surgery, one surgeon must maintain the reduction while the other performs fixation. Although there are various fixation methods for pediatric supracondylar humerus fractures in the literature, the reduction maneuver is widely utilized [19]. There are many options for the surgical treatment of Gartland type 3–4 supracondylar humerus fractures. Discussions regarding the potential risk of ulnar nerve injury in case of medial pinning and the insufficiency of lateral pinning alone in providing stability compared to cross pinning are still ongoing [20,21]. Li et al. performed a medial incision and inserted one K-wire from the medial aspect and two K-wires from the lateral aspect, and they did not report any complications such as ulnar nerve injury, compartment syndrome, or varus deformity during the follow-up of 83 patients >10 years, and they obtained satisfactory functional and cosmetic outcomes [22]. Lateral pinning was reportedly performed in 48 patients and cross pinning in 39 patients following closed reduction, and cross pinning reportedly provided a more rigid fixation [23]. In this study, we preferred the fabric adhesive bandage fixation method. We first performed pinning in a lateral configuration and then moved away from the ulnar nerve tunnel in the medial aspect under fluoroscopy guidance, as this was safer for the ulnar nerve and provided sufficient biomechanical stability. 

The advantages and disadvantages of open reduction and percutaneous pinning with closed reduction are still debated. Despite these discussions, closed reduction and pinning are still the most commonly used methods [24,25,26,27]. Flynn et al. conducted a study on 52 patients and obtained satisfactory results (98%) according to the current criteria used for the follow-up of supracondylar humerus fractures [28]. Conversely, Tomori et al. asserted that mini-open reduction from the anterior aspect provided better cosmetic and functional outcomes than the standard open reduction [29]. Dabash et al. conducted a study on 17 patients and argued that screw fixation with a medial incision was a stable method to achieve early motion [30]. We preferred closed reduction and percutaneous pinning in all 46 patients and obtained excellent results in 95.6% of the patients. Poor functional and cosmetic outcomes (2.2%) were reported in one of our patients who had heterotopic ossification, as the patient had multiple traumas due to a traffic accident.

Pinning and reduction procedures are performed by different surgeons in most of the aforementioned treatments. Moreover, continuing the reduction procedure for a long time is tiring and can lead to complications such as loss of reduction and prolonged surgery. In a study by Hae et al., the mean duration of surgery was 32 ± 17 min [31]. Naik et al. reported that they performed lateral pinning in only 28.3 ± 1.6 min and cross pinning in 30 ± 3.6 min [32]. In this study, we performed fluoroscopy-guided reduction and plaster fixation procedures on all patients under general anesthesia within a very short period of 8.1 ± 3.9 min. In addition, crossed K-wire configuration was completed in 7.9 ± 1.4 min after sterilization of the elbow area following reduction. There are not many sources in the literature relating to the reduction and pinning method applied by a single surgeon.

In a study by Pavone et al. similar outcomes were reported with either by crossed or lateral pinning of supracondylar humerus fractures in children [33]. Gopinathan et al. reported that Baumann’s angle was 76.05° when a configuration of three parallel K-wires was used and 73.9° when a divergent K-wire configuration was used [34]. A previous study reported that the carrying angle was 10.63° in the elbow on which a parallel K-wire configuration was applied and 9.21° in the intact elbow, but it was 10.82° in the elbow on which a divergent K-wire configuration was applied and 10.36° in the intact elbow [34]. Compared to the present study, Baumann’s angle was 73.9 ± 3.3° in the affected elbow and 73.1 ± 2.2° in the intact elbow at the six month follow-up, whereas the carrying angle was 7.4 ± 2.8° in the affected elbow and 6.9 ± 1.2° in the intact elbow, which are all consistent with those in the literature.

Iatrogenic nerve injury has an incidence rate of 2–4% depending on the fracture type, surgical treatment method, surgeon’s experience, and pinning configuration in the applied technique [35]. Lee et al. conducted a prospective and nonrandomized study on 291 pediatric patients and did not find a difference between using one medial-entry pin with two lateral-entry pins and only lateral-entry pins in terms of ulnar nerve complications [36]. On the contrary, we used one medial-entry and one lateral condylar-entry pin in all patients. There is also an alternative method of fixation of supracondylar humerus fractures in the prone position [37]. In this technique the patient is placed in prone position under general anesthesia and the affected arm hung over the edge of the table. This technique is reported as safe in patients with severe soft tissue swelling and unstable fractures unable to achieve acceptable reduction. We did not need this prone technique because of its difficulties encountered with positioning of the patient and closed reduction was achieved in all patients. As the risk of ulnar nerve damage is increasing with a closed medial K-wire insertion, we used lower degrees of elbow flexion and the skin on the medial epicondyle was moved posteriorly as much as possible while fixing the medial condyle to prevent anterior subluxation of the ulnar nerve. One patient among the 46 patients developed ulnar nerve neuropraxia, which was entirely resolved within three months.

Study limitations include the small number of patients and the retrospective nature of the study. The effectiveness of this technique would be further seen with additional prospective and randomized studies with larger patient groups. 

## 5. Conclusions

Intraoperative preservation of reduction and the use of a crossed configuration of one medial and one lateral K-wire was found to be a safe and applicable method in pediatric supracondylar humerus fractures. Further, the mean duration of this procedure was significantly lower than that of the other procedures reported in the literature.

## Figures and Tables

**Table 1:** Flynn criteria.

	Functional Loss of Range of Motion	Cosmetic Change in the Carrying Angle
Perfect	0–5°	0–5°
Good	6–10°	6–10°
Moderate	11–15°	11–15°
Poor	>15°	>15°

**Table 2:** Demographic distribution.

	*n*	Mean ± SD	Median	Min–Max
Age	46	7.1 ± 3.4	6	2–16
Length of hospital stay (days)	46	4.3 ± 3.9	4	1–29
Duration of reduction and plaster fixation (min)	46	8.1 ± 3.9	6.5	3–18
Duration of surgery (min)	46	7.9 ± 1.4	8	6–12
Duration of follow-up (weeks)	46	48.1 ± 14.3	46	20–82
ROM difference, intact vs. fractured (degrees)	46	0.59 ± 2.8	0	0–16

ROM: range of motion, SD: standard deviation.

**Table 3:** Evaluation of the functional and cosmetic outcomes of the patients.

		*n*	%
Sex	Male	32	69.6
	Female	14	30.4
Side	Right	26	56.5
	Left	20	43.5
Functional outcome	Poor	1	2.2
	Excellent	44	95.6
	Moderate	1	2.2
Cosmetic outcome	Poor	1	2.2
	Excellent	44	95.6
	Moderate	1	2.2

**Table 4:** Radiological evaluation of the intact side and fractured elbow at six months.

		Intact Side	Side with Fracture	
	*n*	Mean ± SD	Mean ± SD	*p*
Baumann’s angle	46	73.1 ± 2.2	73.9 ± 3.3	0.069
Carrying angle	46	6.9 ± 1.2	7.4 ± 2.8	0.303
